# Gelsiminum Sempervirens in Neuralgias

**Published:** 1875-12

**Authors:** 


Gelseminum Sempervirens in Neuralgias.—In the latest number of the *Centralblatt fur Medic. u. Wissenschaft*, Dr. Jurasz, assistant physician Polyklinik, Heidelberg, reports the following cases of neuralgia treated with the tinct. gelsemini, with most excellent results:

Case I.—Robust man, æt. 30; suffering for a week with neuralgia of the first branch of the trigeminus, right side; was treated with quinine, etc., internally; also with various external applications, as ung. veratr. All in vain, however. Was put upon tinct. gelsemini, five drops three times a day; complete cure in three days.

Case II.—Brachial neuralgia of one and a half year's standing in a robust seamstress, æt. 30. At first was treated with liniments, potass. iod., etc., literally; all stopped; ordered tinct. gelsemini five drops three times daily; radical cure in six days.

Case III.—Old man, 64 years; neuralgia of supra-orbital nerve. Tinct. gelsemini, ten drops three times daily; complete cure in four days.

Case IV.—Neuralgia of the first and second branch of trigeminus, both sides, in a robust woman, æt. 38. Tinct. gelsemini, five drops three times daily; cured in twenty-four hours.

Case V.—Ischias of right side, very severe, in an old man 64 years of age; could not use his lower limbs in consequence, and compelled to keep his bed; all treatment ineffectual. Was ordered tinct. gelsemini, eight drops three times daily; in fifteen days he was able to walk about; cure completed by electricity and warm baths.

The doctor also states that in hemicrania and muscular rheumatism, he has found it ineffectual.—*Cin. Lancet and Observer.*

